# Regulation of Stress-Induced Immunosuppression in the Context of Neuroendocrine, Cytokine, and Cellular Processes

**DOI:** 10.3390/biology14010076

**Published:** 2025-01-15

**Authors:** Evgenii Balakin, Ksenia Yurku, Mark Ivanov, Alexander Izotov, Valeriya Nakhod, Vasiliy Pustovoyt

**Affiliations:** 1 Federal Medical Biophysical Center of Federal Medical Biological Agency, 123098 Moscow, Russia; 2 V.N. Orekhovich Research Institute of Biomedical Chemistry, Pogodinskaya Str. 10, Bldg. 8, 119121 Moscow, Russia

**Keywords:** stress-induced immunosuppression, neuroendocrine regulation, cytokine imbalance, immune dysfunction, regulatory mechanisms, chronic stress

## Abstract

Understanding the regulatory mechanisms of stress-induced immunosuppression and developing reliable diagnostic methods are important tasks in clinical medicine. This will allow for the development of effective strategies for the prevention and treatment of conditions associated with immune system dysfunction induced by chronic stress. The purpose of this review is to conduct a comprehensive analysis and synthesis of existing data on the regulatory mechanisms of stress-induced immunosuppression. The review is aimed at identifying key neuroendocrine, cytokine, and cellular processes underlying the suppression of the immune response under stress. This study involved a search of scientific literature covering the neuroendocrine, cellular, and molecular mechanisms of stress-induced immunosuppression regulation, as well as modern methods for its diagnosis. Major international bibliographic databases covering publications in biomedicine, psychophysiology, and immunology were selected for the search. The results of the analysis identified key mechanisms regulating stress-induced immunosuppression. The reviewed publications provided detailed descriptions of the neuroendocrine and cytokine processes underlying immune response suppression under stress. A significant portion of the data confirms that the activation of the hypothalamic–pituitary–adrenal (HPA) axis and subsequent elevation of cortisol levels exert substantial immunosuppressive effects on immune cells, particularly macrophages and lymphocytes, leading to the suppression of innate and adaptive immune responses. The data also highlight the crucial role of cortisol and catecholamines (adrenaline and noradrenaline) in initiating immunosuppressive mechanisms under chronic stress.

## Simple Summary

Understanding the regulatory mechanisms of stress-induced immunosuppression and developing reliable diagnostic methods are important tasks in clinical medicine. This will allow for the development of effective strategies for the prevention and treatment of conditions associated with reduced immune protection against chronic stress. The purpose of this review is to conduct a comprehensive analysis and synthesis of existing data on the regulatory mechanisms of stress-induced immunosuppression. The review is aimed at identifying key neuroendocrine, cytokine, and cellular processes underlying the suppression of the immune response under stress.

## 1. Introduction

The immune system is a highly complex network of cells, cytokines, and signaling pathways that maintain host defense and homeostasis. Among the key immune cells, T cells and B cells play a central role in adaptive immunity, with T cells mediating cellular responses and B cells producing antibodies to target pathogens. Innate immune cells, such as macrophages and dendritic cells, act as the first line of defense by recognizing and phagocytosing pathogens, presenting antigens, and producing cytokines to modulate immune responses. Natural killer (NK) cells contribute to the elimination of virus-infected and tumor cells, while microglia serve as resident immune cells in the central nervous system, involved in neuroinflammatory processes. Cytokines, as essential mediators of immunity, are categorized into pro-inflammatory (e.g., IL-6, TNF-α, and IL-1β), anti-inflammatory (e.g., IL-10), and regulatory cytokines that govern the Th1/Th2 balance, influencing cellular and humoral immunity. Chronic stress disrupts this intricate balance by altering cytokine production and immune cell function, shifting the immune response toward either heightened inflammation or immunosuppression. Understanding these fundamental components and their dysregulation under stress is critical for elucidating the pathophysiological mechanisms linking chronic stress to immune dysfunction and associated diseases [1,2,3]

Modern studies confirm the key role of stress as a potent modifying factor that can affect all physiological systems of the body, including the immune system [4,5]. One of the most significant and simultaneously adverse effects of chronic stress is stress-induced immunosuppression, which results from a complex interplay of neuroendocrine, cytokine, and cellular changes [6,7]. These changes suppress the immune response and create a predisposition toward the development of infectious and chronic inflammatory diseases [8,9].

The foundation of stress-induced immunosuppression is in intricate neuroendocrine mechanisms, including the activation of the hypothalamic –pituitary–adrenal (HPA) axis and the sympathetic nervous system, leading to the release of stress hormones such as cortisol, adrenaline, and noradrenaline [10,11,12,13]. Elevated cortisol levels exert immunosuppressive effects by reducing the number of active lymphocytes and inhibiting cytokine production, thereby suppressing both innate and adaptive immune responses [14,15].

Previous research has also demonstrated that chronic stress can induce cytokine imbalances characterized by the increased production of pro-inflammatory cytokines, such as interleukin-6 (IL-6), and decreased levels of anti-inflammatory interleukins, such as interleukin-10 (IL-10) [16]. This imbalance exacerbates immune dysfunction [17,18]. The resulting state contributes to impaired interactions between immune system cells and fosters chronic inflammation, which can subsequently exert negative effects on the functioning of internal organs and systems [19].

**Figure 1:**
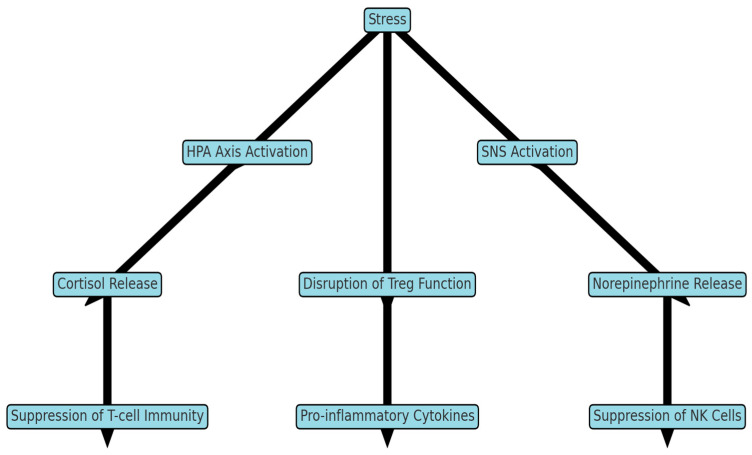
Schematic illustrating the key mechanisms of immune response suppression in chronic stress.

Chronic stress has a significant impact on resident immune cells in the brain. In particular, mast cells and microglia are activated in response to stress signals, leading to the development of neuroinflammation. At the same time, activated microglia begin to secrete pro-inflammatory cytokines such as interleukin-1 beta (IL-1β), tumor necrosis factor-alpha (TNF-α), and IL-6, which exacerbates inflammatory processes in the brain. Simultaneously, mast cells secrete histamine and other inflammatory mediators that disrupt the barrier function of the blood–brain barrier (BBB) and increase the permeability to proinflammatory factors (Figure 1). These processes together contribute to the chronic activation of the brain immune system, which underlies the pathogenesis of neurodegenerative diseases and stress-induced psychiatric disorders [20,21].

The reduction in the body’s protective functions under stress is also associated with changes in the microbiota composition and function, which plays an important role in regulating the immune response [22,23]. Chronic stress causes an imbalance in the interaction between inflammatory mediators and cell populations, including decreased NK-cell and lymphocyte activity, resulting in an impaired coordination of the innate and adaptive immune response [24].

The diagnosis of stress-induced immunosuppression remains a complex task, as it requires methods that enable a comprehensive assessment of the immune system state and stress hormone levels, and the identification of molecular and cellular markers reflecting the degree of immune suppression [25,26]. Currently, biomarkers such as saliva and blood cortisol levels, circulating lymphocyte counts, and cytokine production are used. However, for an accurate diagnosis and the development of effective correction methods, a deeper understanding of the regulatory mechanisms underlying stress-induced immunosuppression is required [27,28,29].

Thus, understanding the regulatory mechanisms of stress-induced immunosuppression and developing reliable diagnostic methods are important tasks in clinical medicine. This will allow for the development of effective strategies for the prevention and treatment of conditions associated with the reduced immune protection against chronic stress.

The purpose of this review is to conduct a comprehensive analysis and synthesis of existing data on the regulatory mechanisms of stress-induced immunosuppression. The review is aimed at identifying key neuroendocrine, cytokine, and cellular processes underlying the suppression of the immune response under stress.

## 2. Materials and Methods

This study involved a search of scientific literature covering the neuroendocrine, cellular, and molecular mechanisms of stress-induced immunosuppression regulation, as well as modern methods for its diagnosis. The search strategy for this review was developed to identify all available and relevant scientific publications addressing the regulatory mechanisms and diagnostic methods of stress-induced immunosuppression. Major international bibliographic databases covering publications in biomedicine, psychophysiology, and immunology were selected for the search. The search was conducted in the following databases: PubMed, Web of Science, Scopus, and the Cochrane Library. These databases provided access to a vast collection of peer-reviewed articles, enabling comprehensive coverage of research on the topic. To identify contemporary and relevant data, the search included publications from 1984 to 2024. This time interval was chosen to ensure access to recent studies conducted using modern methodologies in molecular biology, biochemistry, and clinical diagnostics.

A list of keywords and phrases reflecting the main concepts related to stress-induced immunosuppression was developed for the search. The list of keywords and phrases included the following: “stress-induced immunosuppression”, “immune regulation under stress”, “neuroendocrine-immune interaction”, “biomarkers of immunosuppression”, and “diagnostic methods for stress-induced immune changes”. These keywords and phrases were used both independently and in various combinations with logical operators (“AND”, “OR”, and “NOT”) to broaden the scope and improve the precision of the search. For example, combinations included expressions such as “stress-induced immunosuppression AND neuroendocrine-immune interaction”, “immune regulation AND biomarkers of stress”, and “diagnostic methods AND stress-induced immune changes”.

To improve the quality of the selection, the following restrictions were applied to the search results: 1. type of publications (peer-reviewed scientific articles, meta-analyses, and clinical studies; conference abstracts, editorials, reviews without primary data, and non-peer-reviewed sources were excluded); 2. language of publications (only publications in English and Russian were included in the review, ensuring data accessibility for analysis while maintaining a high level of quality and relevance of sources); 3. availability of full texts (priority was given to articles with full-text access for a more detailed analysis of the methods and data presented in the publication).

**Search Stages**

The search was conducted in several stages. In the first stage, a preliminary search using primary keywords was performed to assess the number and relevance of publications for each of the stated topics. In the second stage, the keywords were refined and supplemented based on the specificity of the database and the thematic focus of the identified publications. Each search in an individual database was documented, including the number of publications found, the combinations of keywords used, and the search dates. In the third stage, filters were applied to the results based on publication type, language, and availability of full text, after which articles were selected for subsequent annotation analysis.

**Relevance Assessment Procedure**

All publications obtained from the search underwent an initial screening based on titles and abstracts. Each publication was evaluated for compliance with the following criteria: (1) presence of a description of mechanisms regulating stress-induced immunosuppression; (2) inclusion of primary data or results supporting the stated conclusions; and (3) methodological transparency and statistical significance of the results. During the secondary screening, the full texts of the publications were reviewed to determine their final relevance. Each selection stage was conducted by two independent reviewers, and, in cases of disagreement, a third expert was involved to resolve disputes.

**Data Collection and Documentation**

A data table was used to document each step, recording information on every study: author, year of publication, article title, database, number of participants (if applicable), primary mechanisms and diagnostic methods, statistical methods, and study conclusions. Reasons for excluding publications from the analysis were also documented, ensuring the transparency and reproducibility of the search and selection process. Thus, the developed and implemented search strategy ensured a reproducible and comprehensive approach to selecting relevant scientific literature for this review.

**Inclusion and Exclusion Criteria**

To ensure the high quality and relevance of data included in the review, strict inclusion and exclusion criteria were developed. These criteria focused on selecting publications that most comprehensively and reliably reflect the mechanisms of regulation and diagnostic methods of stress-induced immunosuppression.

**Inclusion Criteria:** study type, study topic, language and accessibility, full text availability, methodological transparency, data quality. The review included original studies that addressed the mechanisms of regulation of stress-induced immunosuppression and its diagnostic methods. Studies with any design, including cohort, cross-sectional, longitudinal, and randomized controlled trials (RCTs), were considered, provided their results were statistically significant and methodologically sound. Publications were included if they provided data on the following aspects: neuroendocrine and cytokine mechanisms associated with the development of stress-induced immunosuppression and interactions between the nervous and immune systems affecting immune regulation under stress. Publications available in English or Russian, with full-text access, were included, allowing for a detailed analysis of all aspects of the studies, including methodology, results, and conclusions. All included publications provided a detailed description of methodology, including sample size, selection procedures, measurement methods, and data analysis techniques. Studies with sufficient statistical power and evidence to support their conclusions were included. Quality assessment considered sample size, analytical methods, presence of control groups, and the presentation of statistically significant results.

**Exclusion Criteria:** publication type, topics unrelated to the review objectives, methodological limitations, insufficient data on diagnostic methods, lack of statistical significance in results, and lack of full-text access. Articles without primary data, including review articles, editorials, letters to the editor, commentaries, and conference abstracts, were excluded. Publications not aligned with the primary focus of the review were excluded. For instance, studies addressing purely psychological or behavioral aspects of stress without analyzing immunological changes or studies focused solely on certain aspects of immunology unrelated to stress-induced changes were excluded. Additionally, studies focusing on the diagnosis of stress states without analyzing immune response parameters were excluded. Publications with insufficient methodological transparency or weaknesses, such as small sample sizes, lack of control groups, or methods incapable of producing statistically significant data, were excluded. Studies that did not provide detailed data on diagnostic methods for stress-induced immunosuppression, such as biomarker analysis, stress hormone levels, or cytokine levels, were excluded. Publications lacking statistically significant results or presenting unreliable conclusions unsupported by sufficient data were excluded. Finally, studies with access limited to abstracts or fragments were excluded, as this did not allow for a comprehensive analysis of the presented data.

**Selection Process and Quality Assessment**

At all stages, publication selection was based on strictly defined criteria, enabling the exclusion of materials that did not align with the research objectives.

The initial selection of publications was conducted through the analysis of titles and abstracts. This step allowed for the exclusion of materials that did not meet the basic inclusion criteria, such as the presence of a description of mechanisms underlying stress-induced immunosuppression. Reviewers assessed the relevance of each publication concerning the stated topic based on its title and abstract. Publications that did not mention key aspects of the research were excluded from further analysis. All publications not meeting these basic criteria were eliminated at this stage. The selection process was conducted by two independent reviewers, with a third expert involved in cases of disagreement for a final decision.

Publications that passed the initial stage underwent full-text analysis. At this stage, methodological transparency and adherence to more detailed inclusion and exclusion criteria were evaluated. Reviewing the full text allowed reviewers to more accurately assess the publication relevance and verify whether the articles contained statistically significant results, justified conclusions, and data meeting the quality criteria. During this stage, study parameters such as study design, sample size, data analysis methods, and evidence level were recorded. Full-text analysis was conducted by two reviewers, reducing subjectivity and improving the reliability of conclusions. A third expert was engaged in cases of discrepancies in final assessments between the reviewers.

After selecting the publications meeting the inclusion criteria, a quality assessment of each study was conducted. Standardized assessment tools were applied, such as the Newcastle–Ottawa Scale for cohort studies and the Cochrane criteria for randomized controlled trials (RCTs). The quality assessment analyzed the methodological rigor of the studies, their reproducibility, sample representativeness, statistical significance of results, and other indicators important for confirming the reliability of the data. For randomized controlled trials, the assessment included parameters such as the presence of randomization, allocation concealment, and blinding, which minimized the risk of bias in results. These factors were crucial in enhancing the evidentiary value and credibility of the findings.

Analysis of the selected process and documenting it ensured a high degree of reproducibility. All selection stages were recorded in a data table (Table 1) that included information on the number of publications excluded at each stage, reasons for their exclusion, and the main parameters of the included studies: authors, year of publication, study design, sample size, methods applied, and evidence level. This structured the data and ensured the transparency of the analysis.

**Table 1:** Characteristics of studies on stress-induced immunosuppression including design, methods, and evidence levels.

Reference	SD	Sample Size	Methods Applied	Evidence Level	Key Findings
[30]	RCT	394 participants, adults	Stress level assessment, cold development, and cortisol and immune cell analysis	Level I	High stress levels increase susceptibility to infections such as colds by suppressing immune functions.
[31]	LS	40 students	Cortisol levels, T-cell function, and antibody levels	Level II-2	Exam stress reduces T-cell function and antibody levels, indicating temporary immune suppression.
[32]	RCT	11 students (exam stress)	Wound healing speed, and cortisol and cytokine analysis	Level I	Stress delays wound healing by suppressing cytokine production and growth factors necessary for tissue regeneration.
[33]	RCT	48 HIV-infected individuals	Stress reduction program, CD4+ T-cell counts, and viral load analysis	Level I	Stress reduction improves immune function and quality of life in HIV patients.
[34]	RCT	200 breast cancer survivors	Yoga sessions, cytokine analysis, and mood and fatigue levels	Level I	Yoga reduces inflammatory markers, improves mood, and alleviates fatigue in post-cancer treatment patients.
[17]	MA	30 years of research (3000+ participants)	Stress levels and immune function overview, and meta-analysis	Level I	Chronic stress is linked to reduced adaptive immune responses, including decreased T-cell and NK-cell function, increasing inflammation and infection risks.
[23]	R	Several microbiota studies	Stress effects on microbiota, and gut–brain axis	Level III	Stress induces gut microbiota dysbiosis, disrupting immune function and exacerbating inflammation along the gut–brain axis.
[18]	R	Literature data	Stress-inflammatory pathways’ interaction, and social signaling	Level III	Chronic stress activates inflammatory pathways through social neurobehavioral mechanisms, increasing depression and inflammation risks.
[35]	R	Literature data	Chronic stress impacts on inflammation and health	Level III	Chronic stress promotes inflammation, potentially leading to cardiovascular and autoimmune diseases.
[36]	R	Literature data	Psychosocial factors affecting the immune system	Level III	Psychosocial factors, such as stress and depression, negatively impact immune function, increasing disease risk.
[8]	R	Literature data	Interaction between nervous, endocrine, and immune systems under stress	Level III	Stress alters immune-related gene expression through neuroendocrine mechanisms, affecting immune responses.
[15]	R	Literature data	Neuroendocrine regulation of immunity via the HPA axis	Level III	Neuroendocrine mechanisms, including cortisol and catecholamines, play a central role in immune suppression under chronic stress.
[9]	R	Literature data	Acute and chronic stress effects on immune functions	Level III	Acute stress can enhance immune responses in the short term, while chronic stress suppresses immune functions, increasing infection risk.
[37]	Ex	Laboratory animals	Cortisol, pro-inflammatory cytokines, and immune cell levels analysis	Level II-1	Acute stress mobilizes immune cells to peripheral tissues, but prolonged exposure depletes resources and reduces immune activity.
[4]	R	Literature data	Acute and chronic stress impacts on immune function	Level III	Acute stress may boost immunity, while chronic stress suppresses immune function, increasing disease risk.
[29]	MA	41 studies (10–500 participants per study)	Analysis of circulating inflammatory markers (CRP and IL-6) under acute stress	Level I	Acute stress temporarily increases inflammatory markers like CRP and IL-6, confirming activation of inflammatory processes during stress.

Note: SD—study design; RCT—randomized controlled Trial; LS—longitudinal study; MA—meta-analysis; R—review; Ex—experimental study.

To ensure consistency and resolve disagreements at each stage of selection and quality assessment, all interim results were discussed among reviewers. If consensus was not reached, an independent third expert was involved. This process improved objectivity and reduced the risk of subjective evaluations, ultimately minimizing potential biases and contributing to the high quality of the analysis.

## 3. Results

The results of the analysis identified key mechanisms regulating stress-induced immunosuppression. The reviewed publications provided detailed descriptions of neuroendocrine and cytokine processes underlying the immune response suppression under stress. A significant portion of the data confirms that the activation of the HPA axis and subsequent elevation of cortisol levels exert substantial immunosuppressive effects on immune cells, particularly lymphocytes and macrophages, leading to the suppression of innate and adaptive immune responses. The data also highlight the crucial role of cortisol and catecholamines (adrenaline and noradrenaline) in initiating immunosuppressive mechanisms under chronic stress.

**Mechanisms of Stress-Induced Immunosuppression**

**Hormonal Response to Stress**

One of the key mechanisms of stress-induced immunosuppression is the hormonal response mediated by the HPA axis, which regulates the secretion of glucocorticoids, primarily cortisol, and catecholamines such as adrenaline and noradrenaline [38,39,40,41,42]. These hormones play an important role in modulating the immune response, adapting the body to stress by activating physiological processes necessary for resource mobilization and protection, while simultaneously exerting potent immunosuppressive effects [15,43,44].

The hippocampus is a key structure that modulates the negative feedback regulation of the HPA axis via glucocorticoid receptors. The activation of these receptors in the hippocampus inhibits the release of the corticotropin-releasing hormone from the hypothalamus and adrenocorticotropic hormone from the pituitary, thereby regulating glucocorticoid levels. This mechanism plays a crucial role in maintaining homeostasis and preventing the excessive activation of the HPA axis [45,46].

Under stress, the hypothalamus is activated and releases corticotropin-releasing hormone (CRH), which stimulates the anterior pituitary. In response, the pituitary secretes the adrenocorticotropic hormone, which subsequently activates the adrenal cortex, leading to increased cortisol secretion. As the primary glucocorticoid in humans, cortisol modulates many bodily systems, including the immune system, by suppressing inflammation and the activity of immune cells [47,48].

Cortisol reduces T-lymphocyte activity and proliferation, suppresses the production of cytokines such as interleukin-2, and decreases the functional activity of natural killer cells, thereby limiting the body’s ability to respond to infectious agents and abnormal cells [14,15]. By suppressing the activation of T-helper and cytotoxic T cells, cortisol disrupts the coordination between innate and adaptive immune responses, resulting in decreased immune activity across all levels [9,44].

Simultaneously with the HPA axis activation, stress activates the sympathetic nervous system, leading to the release of catecholamines such as adrenaline and noradrenaline. These hormones facilitate short-term resource mobilization by increasing heart rate and blood pressure and activating peripheral vascular responses. However, studies have shown that chronic stress and persistently high catecholamine levels suppress cellular immunity, cause imbalances in cytokine profiles, and reduce the immune system’s ability to combat pathogens [8,49].

The stress-induced activation of the sympathetic nervous system and subsequent catecholamine release modulate macrophage activity by engaging β2-adrenergic receptors, leading to a reduced phagocytic ability and suppressed production of pro-inflammatory cytokines like IL-1β and TNF-α. This dual action mitigates inflammation to limit tissue damage while concurrently diminishing the host’s capacity for rapid immune defense against pathogens, promoting an immunosuppressive state. The prolonged elevation of cortisol levels during chronic stress can initially suppress inflammation [18]. However, glucocorticoid resistance often develops, leading to the increased production of pro-inflammatory cytokines such as IL-6, which contribute to chronic inflammation and conditions like rheumatoid arthritis and type 2 diabetes [19,29].

Feedback mechanisms play an important role in stress-induced immunosuppression. Under normal conditions, elevated cortisol levels activate glucocorticoid receptors in the hypothalamus and pituitary, reducing HPA axis activity. However, under chronic stress, this regulation is disrupted, leading to increased cortisol secretion and sustained immune suppression. This is supported by data showing that patients with chronic stress exhibit elevated cortisol levels and decreased immune indicators, such as NK-cell activity and cytotoxic T-lymphocyte levels [17,25].

Thus, the hormonal response to stress, including the activation of the HPA axis and the sympathetic branch of the autonomic nervous system, plays a central role in the mechanism of stress-induced immunosuppression. Chronic stress causes an imbalance in the interaction between inflammatory mediators and cell populations, including decreased NK-cell and lymphocyte activity, resulting in an impaired coordination of the innate and adaptive immune response [20].

**Adrenaline Surge and Sympathetic Activation in Stress-Induced Immunosuppression Mechanisms**

Sympathetic activation and adrenaline release are crucial mechanisms by which the body responds to stress, mobilizing resources for an immediate threat response [50]. Under stress, the sympathetic division of the autonomic nervous system is activated, leading to the release of catecholamines, including adrenaline and noradrenaline, from the adrenal glands and nerve endings. These hormones, which are central to the “fight or flight” response, induce various physiological changes, including increased heart rate, blood pressure, and blood flow to skeletal muscles [10,11,12,13,51,52].

Adrenaline and noradrenaline also modulate immune system functions, including the suppression of certain responses, which explains the development of stress-induced immunosuppression. Adrenaline interacts with adrenergic receptors on the surface of immune cells, reducing the proliferative activity of lymphocytes and impairing T-helper activation, thereby limiting the body’s ability to mount a full immune response [9,53]. Studies have demonstrated that the activation of β2-adrenergic receptors on immune cells reduces the production of pro-inflammatory cytokines, such as interleukin-12 (IL-12), while increasing the synthesis of anti-inflammatory cytokines, such as IL-10 [54,55].

Catecholamines, by activating β-adrenoreceptors on hematopoietic cells in the bone marrow, enhance the proliferation and mobilization of myeloid suppressor cells. These cells suppress the adaptive immune response by inhibiting the function of T-lymphocytes and antigen-presenting cells through the production of arginase-1, reactive oxygen species, and other metabolites. Thus, catecholamines play a key role in immune suppression under chronic stress, enhancing both peripheral immune suppression and the reduced ability to fight infections [56,57].

Noradrenaline acts on immune cells through β- and α-adrenergic receptors, which are present on the surface of T- and B-lymphocytes, macrophages, and NK cells [54]. The activation of β2-adrenergic receptors on immune cells reduces their proliferation and functional activity. This effect is particularly pronounced with chronic sympathetic activation, explaining the suppression of immune responses in individuals experiencing prolonged stress [55]. Specifically, noradrenaline decreases the production of pro-inflammatory cytokines such as IL-12 and IFN-γ, weakening the body’s anti-infective and anti-tumor responses [58,59,60].

Chronic exposure to noradrenaline also increases levels of anti-inflammatory cytokines, such as IL-10, which, in turn, suppresses the activity of type 1 T-helper cells and macrophages. This creates an imbalance between pro- and anti-inflammatory cytokines, impairing the normal functioning of both innate and adaptive immune responses [5,8]. Numerous studies confirm that the adrenaline release in response to stress leads to elevated levels of interleukin-6 (IL-6) and TNF-α, causing chronic inflammation and predisposing individuals to various diseases, including autoimmune and inflammatory disorders [8,29,58,60].

Sympathetic activation also triggers the migration of immune cells from the bloodstream to organs and tissues such as the skin and mucosa, preparing the body to defend against external agents. However, with chronic stress exposure, these protective functions are suppressed, as adrenaline and noradrenaline reduce NK-cell activity, increasing the susceptibility to viral infections and diminishing the body’s ability to eliminate atypical and malignant cells [61,62,63].

Chronic stress is also associated with the disruption of circadian rhythms that regulate the activity of the sympathetic nervous system and the HPA axis. Stress-induced circadian dysfunction leads to the altered secretion of cortisol and norepinephrine, which impairs the adaptive immune response and contributes to disease progression. For example, studies by Dumbell et al. [64] show that the circadian clock controls the GGA axis activation and cortisol release, affecting the body’s ability to maintain homeostasis under conditions of stress.

Another significant mechanism of immunosuppression during prolonged sympathetic activation is the disruption of tissue barrier functions, such as those of the mucosal membranes, which play a critical role in protecting the body from pathogens. Under the adrenaline and noradrenaline influence, immunoglobulin production, particularly immunoglobulin A (IgA), decreases, weakening the barrier function of mucosa and increasing the risk of pathogenic microorganisms entering the body [5,65].

Sympathetic activation is also associated with the increased expression of adhesion molecules on the endothelium, facilitating the leukocyte migration into tissues. However, under chronic stress, this process becomes impaired, and immune cells respond less effectively to inflammatory signals, subsequently weakening the innate immune response [8,65]. This mechanism further underscores the negative adrenaline and noradrenaline impact on the immune system under chronic stress, explaining the increased susceptibility to infectious and chronic diseases [66,67,68,69,70]. Noradrenaline also affects the gut microbiota, disrupting the balance between various bacterial species. It is known that a microbiota imbalance influences the immune status, as the gut microbiota participates in the regulation of systemic immune responses. Under chronic stress, noradrenaline reduces the population of beneficial bacteria and increases pathogenic strains, potentially exacerbating inflammatory processes and compromising gut barrier function [23,71]. This effect highlights the importance of sympathetic activation in the development of stress-induced immunosuppression, especially in conditions of prolonged stress. Overall, the adrenaline release and prolonged activation of the sympathetic nervous system during stress exert multi-level effects on the immune system, disrupting the balance of pro-inflammatory and anti-inflammatory cytokines, reducing NK-cell activity, and suppressing the protective functions of mucosal barriers. These changes emphasize the importance of understanding the mechanisms of sympathetic activation and its consequences for developing approaches to mitigate and prevent stress-induced immunosuppression [17,18].

**Serotonin Release and Serotonergic Activation in the Mechanisms of Stress-Induced Immunosuppression**

Serotonin, or 5-hydroxytryptamine (5-HT), is a critical neurotransmitter that plays a key role not only in mood regulation and cognitive functions but also in immune response modulation [41,72,73,74]. Serotonergic activation under stress, accompanied by increased serotonin levels in the blood and tissues, exerts multi-level effects on the immune system by modulating its activity and contributing to the development of stress-induced immunosuppression [75,76]. Most of the serotonin in peripheral tissues is regulated by the gut microbiota, which produces approximately 90% of the body serotonin, with the remainder synthesized in the brain and nervous system [77]. In response to stress, the activation of serotonergic neurons and serotonin release enhance the interaction between the central and peripheral nervous systems, influencing HPA axis activation and modulating cortisol secretion and other stress hormones [78,79]. Serotonin modulates the activity of immune cells, particularly T-lymphocytes, macrophages, and natural killer (NK) cells, which can lead to the suppression of both innate and adaptive immune responses [80].

The mechanism of serotonin’s influence on immune cells involves various 5-HT receptors, especially 5-HT1A, 5-HT2A, and 5-HT3, expressed on the surface of T cells, macrophages, and other immune elements [81]. The activation of these receptors by serotonin can modulate cytokine production, altering the balance between pro-inflammatory and anti-inflammatory mediators. Specifically, studies have shown that the activation of 5-HT1A and 5-HT2A serotonin receptors suppresses the production of interferon-gamma (IFN-γ) and TNF-α, weakening the cellular immune response and promoting the development of immune dysfunction under chronic stress [75,76]. Additionally, serotonergic activation increases the production of anti-inflammatory cytokines, such as IL-10, which suppresses the inflammatory response and inhibits the activity of macrophages and T-helper cells. This cytokine profile shift contributes to stress-induced immunosuppression, reducing the body’s ability to respond to pathogenic organisms and abnormal cells [77,82].

In addition, it is important to highlight the proven influence of serotonin on the interaction between the gut microbiota and the immune system. Stress-induced changes in serotonin levels can disrupt the composition of the microbiota, which, in turn, affects immune responses. Under chronic stress, a reduction in beneficial bacteria such as Lactobacillus and an increase in opportunistic microorganisms have been observed, leading to impaired gut barrier function and elevated levels of inflammatory markers [83]. These changes contribute to the development of systemic inflammation and create conditions for chronic immunosuppression [71].

The role of serotonin in the activity of NK-cell modulating is also significant. Serotonin can suppress the cytotoxic activity of NK cells through receptor-mediated mechanisms, reducing their ability to eliminate infected and tumor cells. Studies indicate that chronic exposure to serotonin decreases the proliferation and functional activity of NK cells, making the body more vulnerable to viral infections and tumor progression [84].

Under chronic stress, serotonin may also affect the synthesis and secretion of cortisol, amplifying its immunosuppressive effects [73,74,85]. Serotonergic activation has been shown to lead to sustained cortisol elevation, further suppressing immune activity, inhibiting T-cell proliferation, and reducing the cytokine production necessary for an effective immune response [86].

Thus, serotonin release and serotonergic activation play a critical role in the mechanisms of stress-induced immunosuppression. By acting through specific receptors on the surface of immune cells, serotonin disrupts the balance between pro-inflammatory and anti-inflammatory cytokine production, reduces NK-cell activity, and impairs gut barrier functions. These effects are exacerbated by chronic stress, rendering the body more susceptible to infections and chronic inflammatory diseases [17,18].

**Dopamine Release and Dopaminergic Activation in the Mechanisms of Stress-Induced Immunosuppression**

Dopamine, as a key central nervous system neurotransmitter, plays a central role in motivation, mood, and behavior regulating, but also exerts significant effects on the immune system. Dopaminergic activation and dopamine release during stress influence the immune response both directly and indirectly by modulating the function of various immune cells and inducing stress-induced immunosuppression [87,88]. This neurotransmitter regulates immune activity through specific dopamine receptors expressed on the surface of immune cells such as T-lymphocytes, B-lymphocytes, and macrophages, establishing a close connection between the nervous and immune systems [89,90].

Under stress conditions, the dopaminergic system is activated, leading to increased dopamine levels in peripheral blood. The activation of dopamine D1 and D2 receptors on the surface of T-lymphocytes has been shown to reduce the proliferative activity and suppress the function of cytotoxic T cells, weakening the adaptive immune response and making the body more susceptible to infections and abnormal cell development [91,92]. Dopamine also affects T-cell polarization, stimulating the development of type 2 T-helper (Th2) cells, which suppresses cellular immune responses while supporting humoral reactions, thereby exacerbating the imbalance within the immune system [93].

Dopaminergic activation contributes to the cytokine profile changes, which is a key mechanism of stress-induced immunosuppression. Specifically, dopamine, acting through D1 receptors, reduces the production of pro-inflammatory cytokines such as IFN-γ and TNF-α, thereby limiting the body’s ability to effectively defend against pathogenic microorganisms [62,63,88]. Simultaneously, dopamine increases the production of IL-10 and other anti-inflammatory cytokines, promoting an immunosuppressive state by disrupting the balance between pro-inflammatory and anti-inflammatory mediators [7,94]. This shift in the cytokine profile suppresses inflammatory reactions but also compromises the body anti-infective and anti-tumor responses [95]. In addition, dopaminergic activation significantly impacts macrophages and other innate immune cells. Dopamine decreases macrophage activity and suppresses their ability to phagocytize pathogens, thereby weakening the body primary immune defense [93].

A critical aspect of dopamine action is its influence on the mucosal surface’s barrier functions, particularly in the gut. Dopaminergic activation is known to impair gut barrier function by reducing the production of IgA and other secretory immune factors, thereby increasing the intestinal permeability to pathogenic microorganisms [96]. Dopamine also regulates the interaction between the nervous system and the gut microbiota, which plays a pivotal role in modulating immune responses. Microbiota imbalances caused by chronic stress and dopaminergic activation lead to alterations in the microbial community composition and a reduction in beneficial bacteria, weakening the immune system and creating conditions conducive to chronic inflammatory diseases [97].

Thus, dopamine release and dopaminergic activation exert complex effects on the immune system, disrupting the cytokine balance, suppressing T-cell and NK-cell activity, reducing gut barrier function, and altering the microbiota composition. These effects are particularly pronounced under conditions of chronic stress, making dopamine one of the key factors involved in the mechanisms of stress-induced immunosuppression. Understanding dopamine’s role in immune response regulation may aid in developing new strategies for preventing and treating stress-induced immune dysfunctions [18,87].

**GABAergic Activation in the Mechanisms of Stress-Induced Immunosuppression**

Gamma-aminobutyric acid (GABA), the primary central nervous system inhibitory neurotransmitter, significantly influences both behavioral and immune responses under stress conditions [85,98,99,100]. GABAergic activation, characterized by increased GABA levels in the synapses and the activation of GABA receptors, not only exerts an inhibitory effect on neuronal networks, reducing anxiety and tension, but also plays a crucial modulatory role in the immune system [101]. This influence is a significant factor in the mechanisms of stress-induced immunosuppression, primarily through its impact on the balance of pro-inflammatory and anti-inflammatory responses and the function of immune cells [102].

The primary mechanism of GABA action involves the activation of GABA-A and GABA-B receptors, inducing the hyperpolarization of neurons and reducing their excitability. However, GABA’s effects extend beyond the central nervous system; GABA receptors are also expressed on immune cells, including T-lymphocytes, macrophages, and dendritic cells, enabling GABA to directly influence immune responses. The activation of GABA receptors on immune cells results in decreased proliferation and functional activity, explaining the suppression of immune responses under stress conditions [103,104].

GABAergic activation leads to the suppression of pro-inflammatory cytokines such as IL-6 and TNF-α, while increasing the production of anti-inflammatory cytokines such as IL-10. This effect contributes to stress-induced immunosuppression, as the reduction in pro-inflammatory cytokines limits the body inflammatory response and its ability to combat infections and tissue damage [101,105]. GABAergic activation has been shown to particularly suppress type 1 T-helper cell function, leading to a decline in cellular immunity and increased susceptibility to viral and bacterial infections [106].

GABA also directly affects NK cells and macrophages, reducing their activity and cytotoxic functions. Studies have demonstrated that GABAergic activation suppresses the production of IFN-γ and other key molecules necessary for the full functionality of NK cells and macrophages, weakening their ability to recognize and destroy pathogens and abnormal cells [107]. This effect significantly diminishes innate immune responses, facilitating the easier invasion and dissemination of pathogens, especially under prolonged stress conditions.

The reduced activity of macrophages and other innate immune cells under GABA’s influence can be attributed to the changes in metabolism and energy states. Through the receptor activation on macrophages, GABA promotes a metabolic shift toward an anti-inflammatory macrophage phenotype (M2), further diminishing their phagocytic ability and defensive functions [107]. While M2 macrophages exhibit anti-inflammatory functions, their defensive role may differ depending on the specific context of chronic stress or pathological conditions.

This phenomenon is supported by studies showing that elevated GABA levels during chronic stress correlate with an increase in anti-inflammatory macrophage populations and a decrease in active phagocytes [103,104].

GABAergic activation also impacts intestinal barrier functions, which are critical to the systemic immune status of the organism. It is known that GABA, by modulating the gut microbiota, reduces the intestinal barrier permeability and weakens the local immune response. This condition, known as leaky gut syndrome, predisposes individuals to systemic inflammation and further immune suppression, as pathogens and toxins can more easily enter the bloodstream and trigger systemic reactions [83,101,108]. Under chronic stress, these changes in gut barrier functions exacerbate immunosuppression, increasing the risk of infections and chronic inflammatory diseases.

Thus, GABAergic activation under stress exerts multi-level effects on the immune system, reducing the activity of T-lymphocytes, macrophages, and natural killer cells, suppressing pro-inflammatory cytokine production, and impairing gut barrier functions. These effects, particularly pronounced during chronic stress, play a crucial role in the mechanisms of stress-induced immunosuppression, weakening the body’s ability to defend against infections and malignant cells [101,107].

**Cytokine Imbalance in the Mechanisms of Stress-Induced Immunosuppression**

Cytokines, as key regulators of the immune response, play a crucial role in maintaining homeostasis and coordinating interactions between innate and adaptive immunity (Table 2). Under stress conditions, the balance between pro-inflammatory and anti-inflammatory cytokines is disrupted, significantly contributing to the development of stress-induced immunosuppression and chronic inflammation [4,5]. Cytokine imbalance weakens the body immune defense, making it more susceptible to infections, inflammatory disorders, and autoimmune diseases [66,109,110,111,112].

Acute stress often triggers a transient increase in pro-inflammatory cytokines (e.g., IL-6, IL-8, and TNF-α) as part of an adaptive immune response. In chronic stress, however, prolonged neuroendocrine activity suppresses these cytokines in some contexts, except where glucocorticoid resistance or other compensatory mechanisms emerge [113,114]. The stress-induced activation of the HPA axis and the sympathetic nervous system stimulates the release of cortisol and catecholamines, which modulate the production of these pro-inflammatory mediators. Studies indicate that elevated IL-6 levels associated with chronic stress can act as mediators of chronic inflammation, contributing to the development of metabolic and cardiovascular diseases while also reducing immune activity [29,115].

Pro-inflammatory cytokines such as IL-6 and TNF-α have a dual role: on the one hand, they trigger protective mechanisms aimed at eliminating pathogens and damaged cells; on the other hand, their persistently high levels during chronic stress deplete immune resources and promote the development of an immunosuppressive state [8,116]. The chronic production of IL-6 and TNF-α leads to the increased expression of adhesion molecules and the accumulation of inflammatory cells in tissues, provoking tissue damage and potentially resulting in autoimmune processes [7,18].

Furthermore, stress-induced immunosuppression is characterized by reduced levels of anti-inflammatory cytokines such as IL-10 and IFN-α. IL-10, an essential modulator of the immune response, prevents excessive inflammation by suppressing the production of pro-inflammatory cytokines and the activity of type 1 T-helper cells (Th1) [95]. Reduced IL-10 levels disrupt the regulation of inflammatory responses, further contributing to chronic inflammation and the immune system imbalance [117,118]. Persistently low IL-10 levels are associated with an increased susceptibility to infections and a diminished ability of the immune system to regulate inflammatory processes effectively [119,120].

A cytokine imbalance during stress also manifests as an altered ratio of type 1 and type 2 T-helper cells (Th1/Th2) [121]. Chronic stress often leads to reduced anti-inflammatory cytokines such as IL-10 and IFN-α, impairing immune regulation. Simultaneously, a Th2 shift occurs, characterized by increased IL-4 and IL-13, which suppresses cellular immunity while promoting humoral responses. Stress triggers a shift toward a Th2-dominated response, which supports humoral immunity through antibody production but weakens the cellular immunity necessary for the protection against intracellular pathogens such as viruses and certain bacteria [122]. This Th2 shift is accompanied by increased levels of interleukin-4 (IL-4) and interleukin-13 (IL-13), suppressing T-cell and macrophage functions and limiting anti-infective activity [17]. Such changes reduce the body’s ability to combat infections effectively and may lead to the chronicity of infections and an exacerbation of immunosuppression.

Research also shows that a cytokine imbalance during stress contributes to the impaired barrier function of mucosal surfaces, particularly in the gut. Pro-inflammatory cytokines such as IL-6 and TNF-α adversely affect gut barrier cells, increasing their permeability and promoting leaky gut syndrome. This allows pathogens and toxins to enter the systemic circulation, causing systemic inflammation and exacerbating immune dysfunction [83,123]. Gut barrier disruption due to a cytokine imbalance plays a significant role in systemic immunosuppression, especially under chronic stress conditions.

Overall, cytokine imbalance is a key mechanism through which stress impacts the immune system. The increased production of pro-inflammatory cytokines and reduced levels of anti-inflammatory mediators disrupt immune homeostasis, weakening the body’s defense mechanisms and increasing the risk of infectious and inflammatory diseases. These effects underscore the need for further investigation into the cytokine imbalance to develop effective methods for preventing and addressing stress-induced immunosuppression [4,18].

Genetic approaches that are based on specific single-nucleotide polymorphisms (SNPs) have demonstrated significant potential in predicting the risk and activity of human diseases [124]. The ability to predict individual changes in immune parameters in response to psychological stressors is crucial for overall health management and holds potential utility in the treatment of stress-related diseases. Since genetic polymorphisms are relatively stable biomarkers, this approach can be leveraged to classify individuals based on their susceptibility to stress-associated immune responses. Furthermore, polymorphisms in genes from various components of the psychoneuroendocrine-immune network may serve as valuable biomarkers for identifying and categorizing these individual immune reactions [125,126,127].

A number of studies have shown that polymorphisms in the serotonin (5-hydroxytryptamine, 5HT) transporter (5HTT) gene, specifically the 5HTTLPR genotypes, influence stress-induced immune and inflammatory responses. Notably, individuals with the SS genotype exhibit heightened IL-1β reactivity, likely driven by the increased sympathetic nervous system activation during acute stress. Additionally, the elevated baseline and stress-related IL-6/IL-10 ratios in SS carriers suggest a chronic pro-inflammatory bias associated with reduced serotonin levels [29,128,129,130].

**Table 2:** Characteristics of studies on stress-induced humoral immunosuppression.

Reference	Participants (Male/Female)	Baseline Age (Mean or Range)	Observation Duration	Biomarker	Measurement	Method	Result	Conclusion
[114]	119 (43/76)	Mean: 70 years	6 years	IL-6	Serum	ELISA	Increased IL-6 levels in caregivers of dementia patients compared to controls.	Chronic stress accelerates age-related increases in pro-inflammatory cytokines like IL-6.
[131]	276 (Gender not specified)	18–55 years	1 year	IL-6	Serum	ELISA	Participants with high stress levels were more likely to develop colds, correlating with elevated IL-6 levels.	Psychological stress enhances susceptibility to upper respiratory infections through immune response modulation.
[17]	18,941 (Meta-analysis)	Varied	Varied	Various (NK cells, T cells, etc.)	Various	ELISA (hs-CRP)	Chronic stress is associated with reduced lymphocyte count and functional activity.	Prolonged stress negatively affects cellular immunity, reducing defensive functions.
[132]	75 (Gender not specified)	Mean: 34 years	6 months	CRP	Serum	ELISA (hs-CRP)	Participants with chronic stress showed significantly higher CRP levels than controls.	Chronic stress promotes systemic inflammation, reflected in elevated CRP levels.
[5]	13 (Gender not specified)	Mean: 55 years	8 weeks	IL-1β	Wound fluid	ELISA	Stress slowed wound healing, accompanied by increased IL-1β levels.	Stress delays tissue healing processes via cytokine modulation.
[133]	40 (20/20)	24–50 years	1 year	TNF-α	Serum	ELISA	Chronic stress participants exhibited significantly higher TNF-α levels than controls.	Chronic stress is linked to elevated pro-inflammatory cytokines like TNF-α.
[29]	199 (102/97)	Mean: 63 years	3 years	CRP	Serum	ELISA (hs-CRP)	Workplace stress correlated with increased CRP levels.	Psychosocial workplace stress contributes to systemic inflammation.
[134]	6425 women	43–70 years	12 years	CRP (mg/L), IL-6 (pg/mL), TNFα-R2 (pg/mL)	Blood, every 4 years	hs-CRP, ELISA	CRP (Q1: 0.36; Q4: 4.55), IL-6 (Q1: 0.58; Q4: 2.61), TNFα-R2 (Q1: 1855; Q4: 3406). Q—quintile	Chronic stress elevates inflammatory markers like CRP and IL-6.
[135]	9275 (4841/4464)	18–49 years	15 years	suPAR (ng/mL)	Blood, single time point	ELISA	Mean suPAR levels: women 2.54, men 2.22; significantly increased under stress. suPAR—soluble urokinase plasminogen activation receptor	Chronic stress is associated with elevated suPAR levels, a disease risk marker.
[136]	1504 (852/652)	35–66 years	5.5 years	CRP, IL-1β, IL-6, TNF-α (pg/mL)	Serum, single time point	hs-CRP, ELISA	CRP: 1.0 (0.5–2.2); IL-1β: 0.5 (0.1–1.9); IL-6: 1.4 (0.6–3.4); TNF-α: 2.8 (1.8–4.5).	Inflammatory cytokines increase under combined psychological and metabolic stressors.
[137]	5810 (3044/2766)	9–21 years	12 years	CRP (mg/L)	Dried blood spot, single time point	ELISA	CRP: under chronic stress: 2.58 (1.14–5.82).	Chronic stress in adolescence significantly increases CRP levels, indicating systemic inflammation.
[138]	400 (200/200)	35–65 years	2 years	CRP, IL-6, TNF-α	Serum	ELISA	Elevated CRP, IL-6, and TNF-α levels correlated with signs of metabolic dysfunction in high-stress participants.	Perceived stress is linked to inflammatory markers and metabolic dysregulation, increasing chronic disease risk.

**Pathophysiological Processes in Acute Stress**

The cellular immune imbalance during acute stress arises because of the body’s rapid and complex response to stressors, involving the activation of the HPA axis and the sympathetic nervous system. These processes lead to the release of cortisol and catecholamines (adrenaline and noradrenaline), which immediately affect various immune cell populations, disrupting their balance and functions. Key changes include the redistribution of bloodstream immune cells, the reduced activity of some cellular types, and the enhanced functions of others, contributing to rapid alterations in cellular immune responses [4,5].

In response to acute stress, the mobilization of neutrophils and monocytes into the systemic circulation is observed, driven by the catecholamine release. These cells form the first line of defense, participating in the rapid elimination of pathogens and damaged cells. Elevated adrenaline and noradrenaline levels facilitate a rapid increase in circulating neutrophils and monocytes, preparing the body for potential infections or tissue injury [139,140,141]. However, prolonged exposure to acute stress may deplete these cell reserves, weakening their functional capacity over time [42,142].

A characteristic manifestation of the cellular immune imbalance during acute stress is the redistribution of lymphocytes. Stress can reduce T- and B-lymphocyte counts in peripheral blood as these cells migrate to lymphoid organs and tissues. This phenomenon is associated with elevated cortisol levels, which promote lymphocyte migration to lymph nodes, the spleen, and other lymphoid tissues, where they remain “on standby” for potential infections [47,132]. This redistribution is thought to help the body prevent excessive inflammation and protect tissues from potential autoimmune responses.

Acute stress also induces significant changes in NK-cell activity, which are key effector cells of innate immunity responsible for destroying viruses and tumor cells. Acute stress temporarily enhances NK-cell activity, boosting anti-infective and anti-tumor defenses. However, prolonged stress exposure can deplete NK-cell functional reserves, reducing their cytotoxic activity and thereby increasing the body vulnerability to viral infections and malignant cells [143,144].

The activation of the sympathetic nervous system and catecholamine release during acute stress also directly affect macrophages, which are key cells in the inflammatory response. Cortisol and catecholamines activate β2-adrenergic receptors on macrophage surfaces, suppressing their phagocytic ability and reducing the production of pro-inflammatory cytokines such as IL-1β and TNF-α [59,66,67,68,69,70]. This process limits inflammation and minimizes tissue damage but simultaneously reduces the body’s ability to mount a rapid defense against pathogens [54,59]. As a result, macrophages are less capable of eliminating pathogens and initiating an inflammatory response, limiting immune reactions and promoting an immunosuppressive state [54]. Thus, while acute stress may reduce the inflammatory response by modulating macrophage activity, prolonged exposure to stress decreases their effectiveness in combating infections.

**Pathophysiological Processes in Chronic Stress**

One of the critical changes observed during chronic stress is the reduced activity of NK cells, which play a pivotal role in antiviral and anti-tumor defense. Chronic stress diminishes NK-cell cytotoxic activity due to the effects of cortisol and catecholamines on their functional capacity [145]. Reduced NK-cell activity increases the body’s vulnerability to viral infections and tumor cells, as these cells lose their ability to effectively recognize and eliminate infected and transformed cells [146]. Studies have shown that patients experiencing chronic stress exhibit decreased NK-cell counts and cytotoxic activity, correlating with an increased risk of infectious diseases and cancer progression [147]. Another important aspect of the cellular immune imbalance during chronic stress is the reduced proliferative activity of T-lymphocytes [148]. Cortisol and catecholamines exert inhibitory effects on T-cell activation and division, limiting their ability to proliferate in response to antigenic stimulation [9]. This imbalance leads to a reduction in the number and functional activity of cytotoxic T-lymphocytes, which play a central role in the cellular immunity and defense against viral and bacterial infections [148]. The decreased T-cell activity under chronic stress makes the body more vulnerable to various pathogens and contributes to the chronicity of infectious processes [17].

Chronic stress also affects the balance between Th1/Th2, causing a shift in the immune response towards a Th2-dominant state [149]. This process is driven by cortisol, which suppresses the Th1 response—essential for cellular immunity—and enhances the Th2 response, which promotes humoral immunity [150]. The shift toward a Th2 response leads to increased levels of IL-4 and IL-10, weakening the cellular immune response needed to combat intracellular pathogens and tumor cells [9,122]. Consequently, the body loses its ability to effectively counter infections and control tumor growth, resulting in weakened anti-infective and anti-tumor defenses [151,152].

Additionally, chronic stress influences the population of regulatory T cells (Treg cells), which play a critical role in maintaining immune homeostasis and preventing autoimmune reactions. Research shows that chronic stress can increase the number of Treg cells, enhancing their immunoregulatory activity and suppressing cytotoxic T cells and NK cells [153]. The heightened activity of Treg cells may contribute to immunosuppression and reduced resistance to infections and tumor development by inhibiting the activity of other immune cells involved in protective responses [18].

The cellular composition imbalance also affects B-lymphocytes, which are responsible for antibody production [148]. Chronic stress decreases the number of activated B-cells, weakens humoral immunity, and limits the body’s ability to produce specific antibodies against pathogens [7,25]. This is supported by evidence showing reduced antibody levels in response to vaccination among individuals experiencing chronic stress, indicating impaired B-cell function and a diminished capacity for an effective humoral immune response [154].

Thus, the cellular immune imbalance during acute stress is characterized by complex changes, including neutrophil and monocyte mobilization, lymphocyte redistribution, the temporary enhancement of NK-cell activity, and the modulation of macrophage function. These changes represent an adaptive response aimed at mobilizing resources for defense. However, under chronic stress, they become a constellation of disruptions, including decreased NK-cell and T-lymphocyte activity, dysregulated macrophage function, heightened Treg cell activity, and weakened B-cell-mediated humoral immunity. These alterations undermine anti-infective and anti-tumor defenses, increasing the risk of infectious and inflammatory diseases [4,143]. These mechanisms highlight the need for further research into the cellular immune imbalance under chronic stress to develop strategies for preventing and addressing stress-induced immune dysfunctions [4,18].

**Changes in the Microbiota Under Stress**

Microbiota changes caused by stress represent a critical mechanism through which stress affects the immune system and overall health. The gut microbiota, consisting of numerous microbial species, plays an essential role in regulating metabolic processes, synthesizing vitamins, and maintaining immune homeostasis. Under normal conditions, the microbiota maintains a balance between beneficial and potentially pathogenic bacteria, ensuring adequate gut barrier function and supporting immune response activation [23,71]. However, stress disrupts this balance, potentially leading to various pathological conditions, including chronic inflammation, reduced immune defense, and an increased risk of infections.

One mechanism by which stress impacts the microbiota is through the activation of the HPA axis and the subsequent elevation of cortisol levels. Cortisol, the primary stress hormone, directly affects the gut microbiota, altering the composition of microbial communities. Specifically, studies have shown that stress reduces the abundance of beneficial bacteria such as Lactobacillus and Bifidobacterium, which are vital for maintaining the immune response and gut barrier function [23,71]. At the same time, stress increases the opportunistic microorganisms populations such as Escherichia coli and Enterobacteriaceae, promoting inflammatory processes and impairing immune regulation [106,155].

Stress also impacts the intestinal barrier, increasing its permeability, which is known in medical literature as “leaky gut syndrome”. An increased intestinal permeability allows bacteria and their metabolic byproducts to enter the bloodstream, triggering systemic inflammation and exacerbating immunosuppression [156]. This process activates the immune system, leading to the release of pro-inflammatory cytokines such as IL-6 and TNF-α, which further aggravate inflammation and disrupt immune homeostasis [157]. The increased permeability of the gut and the entry of bacterial antigens into the bloodstream can act as triggers for chronic inflammatory diseases such as inflammatory bowel disease (IBD) and irritable bowel syndrome (IBS) [23,158].

Chronic stress also affects the metabolic processes of the gastrointestinal microorganisms. Stress has been shown to alter the production of short-chain fatty acids (SCFAs) such as butyrate, propionate, and acetate, which play critical roles in maintaining gut barrier function and regulating immune responses. SCFAs serve as an essential energy source for intestinal epithelial cells and possess anti-inflammatory properties. During stress, particularly chronic stress, SCFA production decreases, weakening barrier function, increasing permeability, and promoting inflammation [159,160]. Impaired SCFA synthesis also reduces the activity of regulatory T cells (Treg), which are crucial for suppressing autoimmune reactions and maintaining immune homeostasis [161].

The activation of the sympathetic nervous system during stress and the release of catecholamines, such as norepinephrine and adrenaline, also have an association with the microbiota composition and its metabolic activity. Catecholamines can modulate the growth and virulence of certain bacteria, enhancing their pathogenic properties and provoking inflammatory processes [162].

Studies have demonstrated that norepinephrine can stimulate the growth and invasiveness of opportunistic bacteria such as Escherichia coli, exacerbating inflammation and disrupting microbiota balance [163].

Furthermore, stress affects the “gut–brain axis”, a bidirectional communication system between the central nervous system and the gut microbiota. Stress-induced changes in the microbiota can influence emotional states and cognitive functions through neuroinflammation and disrupted neurotransmitter synthesis, such as serotonin, a significant portion of which is produced in the gut. Impaired serotonin synthesis during stress impacts mood and behavior, creating a “vicious cycle” in which stress induces microbiota changes, which, in turn, exacerbate the stress response [157,164].

Thus, stress-induced changes in microbiota have multi-level impacts on the immune system and overall physiological status. The reduction in beneficial bacteria, increased gut barrier permeability, decreased SCFA synthesis, and activation of the gut–brain axis contribute to systemic inflammation, impaired immune homeostasis, and an elevated risk of chronic inflammatory diseases. The gut–brain axis is a dynamic bidirectional communication system that operates under both physiological and pathological conditions. During stress, the dysregulation of the gut–brain axis contributes to systemic inflammation, distinguishing it from its normal physiological activation, which supports homeostasis [23].

These findings underscore the importance of targeting the microbiota for preventing and addressing stress-induced immune dysfunctions [23,71].

## 4. Discussion

The results of numerous studies confirm that stress-induced immunosuppression significantly affects the immune system functional state, causing imbalances between cellular and humoral immunity, the suppression of innate and adaptive immune responses, and the disruption of regulatory mechanisms. These effects, which underlie the immunosuppressive state, become particularly pronounced under chronic stress, characterized by the sustained activation of the HPA axis, regulating the release of glucocorticoids, and the sympathetic division of the autonomic nervous system, leading to catecholamine release [4,5].

One of the key consequences of stress-induced immunosuppression is the increased susceptibility to infections. The weakened immune response results in the reduced activity of NK cells, macrophages, and cytotoxic T-lymphocytes, which are central to the defense against viral and bacterial pathogens. Research shows that individuals experiencing prolonged stress exhibit higher rates of infectious diseases, such as respiratory infections, and a slower recovery from infections [8,9]. These findings highlight the long-term impact of stress on the immune system, reducing its ability to protect the body from pathogens.

Additionally, stress-induced immunosuppression leads to impaired regenerative processes and delayed wound healing. Chronic stress suppresses growth factor production, reduces fibroblast activity, and disrupts angiogenesis, impeding normal tissue repair and increasing healing times [165,166]. This is particularly dangerous for individuals with chronic conditions requiring regular tissue regeneration and underscores the need for strategies to prevent stress-induced impairments in wound healing.

Stress is also associated with an increased risk of oncological diseases, attributed to the reduced immune surveillance of atypical cells and chronic inflammation. Since NK cells and cytotoxic T-lymphocytes play critical roles in eliminating tumor cells, their suppression due to stress promotes tumor growth and metastasis. Furthermore, elevated levels of pro-inflammatory cytokines, such as IL-6 and TNF-α, drive chronic inflammation, creating a favorable environment for tumor cell growth and promoting the angiogenesis necessary for their vascularization [54,167]. These findings emphasize that stress not only reduces the anti-tumor activity of the immune system but also facilitates carcinogenesis, particularly under prolonged stress conditions.

Thus, stress-induced immunosuppression leads to multifaceted changes in the immune system, exerting long-term effects on its protective functions. These include the increased susceptibility to infectious diseases, impaired regenerative processes, and an elevated risk of oncological diseases, as confirmed by numerous clinical and experimental studies [4,5]. These findings highlight the necessity of a comprehensive approach to preventing and addressing stress-induced immunosuppression aimed at maintaining immune homeostasis and reducing the risk of adverse stress-related outcomes.

## 5. Conclusions

Stress affects the immune system functional state, causing imbalances between pro-inflammatory and anti-inflammatory processes, suppressing the activity of the innate and adaptive immunity, and disrupting the gut microbiota. These changes underlie stress-induced immunosuppression, increasing the vulnerability to infections, delaying regenerative processes, and contributing to carcinogenesis. Studies confirm that chronic stress reduces the activity of natural killer cells, T-lymphocytes, and macrophages, while increasing levels of pro-inflammatory cytokines such as IL-6 and TNF-α. This supports chronic inflammation and contributes to oncological diseases. The dysregulation of the immune system is exacerbated by changes in the microbiota, affecting the synthesis of short-chain fatty acids and further disrupting immune processes.

These mechanisms prove the need for integrative approaches to diagnose and prevent stress-induced immunosuppression. The use of biomarkers, including cortisol levels, pro-inflammatory cytokines, and immune cell activity, combined with microbiota evaluation, allows for a more precise characterization of immune response impairment and the adaptive capacity. Consequently, future research should focus on developing comprehensive prevention and treatment strategies, including microbiota correction, stress reduction interventions, and the maintenance of immune homeostasis. These approaches are critical for preventing stress-related pathologies and improving quality of life.

## Data Availability

The data are contained within the article.
